# Association of childhood out-of-home care status with all-cause mortality up to 42-years later: Office of National Statistics Longitudinal Study

**DOI:** 10.1186/s12889-020-08867-3

**Published:** 2020-05-20

**Authors:** Emily T. Murray, Rebecca Lacey, Barbara Maughan, Amanda Sacker

**Affiliations:** 1 Department of Epidemiology & Public Health, University College London, 1-19 Torrington Place, LONDON, WC1E 6BT United Kingdom; 2 MRC Social, Genetic and Developmental Psychiatry Centre, King’s College London, 16 De Crespigny Park, London, SE5 8AF UK

**Keywords:** Mortality, England, Wales, Longitudinal, Looked after children, Child welfare, Foster care, Life course

## Abstract

**Background:**

The adverse life-long consequences of being looked-after as a child are well recognised, but follow-up periods for mortality risk have mostly ended in young adulthood and mortality suggested to differ by age of placement, gender and cohort in small samples.

**Methods:**

Data on 353,601 Office for National Statistics Longitudinal Study (LS) members during census years 1971–2001, and Cox proportional hazards regression models with time-varying covariates (age as the timescale), were used to examine whether childhood out-of-home care was associated with all-cause mortality until the end of 2013. After adjusting for baseline age and age^2^, gender, born outside the United Kingdom, number of census observations in childhood and baseline census year we tested whether mortality risk varied for those in care by age, gender and baseline census year, by separate assessment of interaction terms. Supplementary analyses assessed robustness of findings.

**Results:**

Adults who had been in care at any census (maximum of two) had an adjusted all-cause mortality hazard ratio 1.62 (95% CI 1.43, 1.86) times higher than adults who had never been in care. The excess mortality was mainly attributable to deaths categorised as self-harm, accidents and mental & behavioural causes. Mortality risk was elevated if the LS member was initially assessed in 1981 or 2001, compared to 1971. There was no significant variation in mortality risk for those in care by age or gender. The main findings were consistent irrespective of choice of comparison group (whole population, disadvantaged population), care placement (residential, non-residential) and age at death (all ages, adulthood only).

**Conclusions:**

In this large, nationally representative study of dependent children resident in England and Wales, those who had been in care during childhood had a higher risk of mortality long after they had left care on average, mainly from unnatural causes. No differences by age or gender were found. Children in care have not benefitted from the general decline in mortality risk over time.

## Background

As of March 2018, the number of children looked-after by local authorities in England and Wales was 75,420 and 6407 respectively; equivalent to 62 and 102 per 10,000 population under aged 18 [1, 2]. Both the number of, and rate of children being looked-after, have increased steadily over the past decade, due to a combination of more children entering, and fewer children leaving, care [1].

The adverse life-long consequences of being looked-after as a child are well recognised. This includes worse physical [3–6] and mental health [3–5, 7–13], lower educational attainment, higher rates of unemployment [3, 5, 10, 11, 13], and less stability of housing after care [3, 5, 10, 11, 13]. A small number of studies have shown that children in care have higher mortality than the general population of children, but follow-up periods have mostly ended in young adulthood [14–16]. Only one study we aware of has examined mortality 30 years after care status assessment, but was restricted to children in residential care in 1971 only [3]. As a consequence of the United Kingdom’s (UK’s) 1989 Children Act recommending that placement priority be given to a child’s extended relatives and friends, the percentage of children in residential care has decreased and children in relative household care increased [17–19]. Therefore, it is important to understand whether the previously observed higher mortality risk for looked-after children in residential settings applies to all looked-after children, extends beyond the early adult years, and has continued in later cohorts, in order to identify and hopefully reduce preventable deaths in this vulnerable population.

In addition, studies showed a weak association between care status and mortality in girls placed in early childhood, but stronger evidence for girls placed in care at later ages and for boys placed at any age, mainly from unnatural deaths [15, 16]. One possible explanation concerns the age and gender differences in resilience, with young girls more likely to be resilient to stressful circumstances than young boys [20]. Another includes children entering care at different ages do so for different reasons, parental abuse and developmental issues more common for entry at younger ages and behavioural issues/delinquency more common at older ages [21]. As well, being in care during later childhood may reflect longer placements and multiple placements, which have been associated with more extensive adult emotional and behavioural issues [13, 22]. It is therefore possible that being in care during later childhood and adolescence may be a marker for later adult mortality risk, particularly from unnatural causes.

Thus, we used data from a large nationally representative longitudinal data set to examine 1) whether children who had been in care have higher mortality up to 42 years later than children had in the general population. We also test whether these associations vary, or are explained, by gender or age and year when first observed. The hypotheses for these tests are that given previous findings of gender and age differences, 2) boys in care will have higher mortality than girls in care; and 3) being first observed in care later in childhood will be associated with higher mortality than care earlier in childhood. Finally, 4) given the introduction and implementation of the UK’s 1989 Children Act, there will be a reduction in mortality for those observed more recently.

## Methods

### Data

The Office for National Statistics (ONS) Longitudinal Study (LS) is a 1% representative sample of the population of England and Wales, drawn initially from respondents to the 1971 census who had been born on one of four birthdays [23]. The LS is updated with new members if they have one of the same four birthdays and are either newly-born or immigrants. Members are followed-up in every subsequent 10-year census and are linked to life events data such as births, deaths, and cancer registrations. In order to only include dependent children who could potentially be placed in non-parental care, the sample for this analysis includes individuals aged less than 18 years, of single marital status, not living alone/independently, and not a visitor in the household/residential setting at each of the censuses 1971, 1981, 1991 or 2001. LS members were including in the analysis sample with data from one or two censuses during childhood. Data from only one census occurred if they were not observed at a census 10 years earlier or later or they no longer met the criteria for a dependent child at the next census. LS variables included in the current study did not have any missing values. The analysis sample consisted of 353,601 members with 61% having one and 39% two observations (Table 1 and Supplementary Table S1).

**Table 1:** Distribution of care status by number of observations in childhood, ONS Longitudinal Study

Number of observations	Care status	N	%
One	Y	3439	1.59
	N	212,224	98.41
Two	Y Y	237	0.17
	Y N	763	0.55
	N Y	2005	1.45
	N N	134,933	97.82
Total		491,539	100.00
N		353,601	

Y: in care; N not in care.

### Age at each census

Analysing repeated measures highlighted some inconsistencies in the recording of age. When cleaning the time variables, we prioritised the reliability of year of birth and year of death data over age at each census (i.e. correcting age at census to year of census – year of birth), unless there was an obvious keying error. This process maintained the intra-individual consistency of the data although it is also possible that some unreliability could have been introduced depending on whether a birthday fell before or after the census date.

### Age and cause of death

Age of death was calculated by ONS from year and month of death data from annual matches of LS members with death certificate data [24]. At the time of analysis, all-cause mortality data were available up to 31st December 2013. Individuals who had emigrated or not died by the end of 2013 were treated as right censored. The ONS previously derived a 20-group cause of death categorisation from underlying cause of death data on death certificates, using the International Classification of Diseases coding in use at the time of death [25]. For comparison with previous literature, we further collapsed these 20 categories into a four category cause of death variable: (1) “Unnatural” (Mental or behavioural, accidents or self-harm), (2) “Circulatory” (Ischemic heart disease, Stroke, Pulmonary disease), (3) “Cancer” (Lung cancer, Other cancers, Benign neoplasms) or (4) “Other” (Infectious & parasitic, diabetes, gastro-intestinal tract disease, liver disease, abnormalities & lab results, muscular diseases, nervous system, genito-urinary, other endocrine, skin disease, other cause).

### Care status

For each census from 1971 to 2001, dependent children were classified into those: i) living with a parent, ii) living with a relative (excluding children or relatives aged < 16) or with unrelated others (formal and informal foster care), or iii) living in residential care (a children’s home or place of detention).

### Covariates

Baseline was the first census the sample member responded, out of the potential census years 1971, 1981, 1991 and 2001. Baseline age, gender, country of birth and census year were investigated as potential confounders. Non-linear baseline age effects were modelled using age and age^2^ terms. Responses on census questions about place of birth were collapsed into born in the UK (0 = England, Wales, Scotland and Northern Ireland) or not (1 = any other country listed). To control for any negative selection bias (members may have survived for longer to be observed twice in childhood), a dummy variable indicated if the LS member had been observed in two censuses while still a dependent child.

### Statistical analysis

All care categories and baseline covariates were compared by mortality status at follow-up using Analysis of Variance (ANOVA) for continuous variables and the chi-square statistic for categorical variables. In addition, for LS members who had died, distribution of cause of death was compared across the two care categories separately. We collapsed the care categories into a binary in care or not variable for the main survival analyses using Cox proportional hazards regression models with time-varying covariates (with age as the timescale) that account for intra-individual correlation [26]. Time at risk was assessed from age at baseline census until age at death or right censoring (i.e. the age they reached the end of follow-up). Models were initially fitted unadjusted (model 1), and adjusted for age and age^2^ at baseline, gender, country of birth, baseline census year and the number of childhood observations (model 2). To check for potential increased risks of mortality for children in care by gender, baseline age or during specific periods, we added interaction terms for gender, baseline age and cohort with care status to the adjusted model in models 3, 4 and 5 respectively.

Supplementary analyses checked the robustness of findings. First, in model SM1 we reduced the reference group to those not in care and living in a socially disadvantaged household since children in care are more likely to be from a disadvantaged family and to be living with a more disadvantaged family while in care [27, 28]. Disadvantage was defined as the head of household being in a routine occupational class according to the National Statistics Socio-economic Classification [29]. Frailty models could not be estimated to assess potential confounding by unobserved individual characteristics because of the large sample size. To assess whether mortality applied to those in residential care and non-residential (models SM2 and SM3), we separately re-analysed the data for those in these care situations. A final analysis removed observations for those who had died before the age of 18 (model SM4), under the assumption that death during childhood was more likely to be a consequence of pre-existing poor health than their experience of care. It has also been suggested that mortality from age 18 reflects difficulties in adapting to independent living [15].

All analyses were carried out using Stata 14 [30].

## Results

Sample characteristics by mortality status at follow-up are shown in Table 2. For those LS members who had died (Table 3, *n* = 8814), a higher proportion of deaths for the non-parental care categories occurred within the “Unnatural” (self-harm, accidents and mental/behavioural) causes of deaths, rather than the cardiovascular, cancer or other categories (*p* < 0.0005).

**Table 2:** Sample characteristics by mortality status at end of follow-up on 31st December 2013, ONS Longitudinal Study

	TOTAL (*n* = 353,601)	Dead (*n* = 8814)	Alive (*n* = 344,787)	*p*-value (difference by death status)
1st observation (baseline)				
Median age (SIQR)	353,601	9.0 (10.0)	7.0 (6.5)	0.0001
Gender (%)				< 0.0005
Male	180,905	5607 (3.10)	175,298 (96.90)	
Female	172,696	3207 (1.86)	169,489 (98.14)	
Country of birth (%)				< 0.0005
UK	338,661	8514 (2.51)	330,147 (97.49)	
Other	14,940	300 (2.01)	14,640 (97.99)	
Census year (%)				< 0.0005
1971	135,810	6631 (4.88)	129,179 (95.12)	
1981	73,667	1428 (1.94)	72,239 (98.06)	
1991	74,440	583 (0.78)	73,857 (99.22)	
2001	69,684	172 (0.25)	69,512 (99.75)	
In Care (%)				< 0.0005
Yes	5681	227 (4.00)	5454 (97.53)	
No	347,920	8587 (2.47)	339,333 (96.00)	
2nd observation				
In Care (%)				< 0.0005
Yes	2242	69 (3.08)	2173 (96.92)	
No	135,696	2421 (1.78)	133,275 (98.22)	
Follow-up				
Median age of death or censoring (SIQR)	353,801	40.0 (37.5)	38.0 (37.5)	0.023

*ONS* Office of National Statistics; *SIQR* Semi inter-quartile range.

**Table 3:** Causes of death by care status, ONS Longitudinal Study

Care in childhood	TOTAL	Unnatural (%)	Circulatory (%)	Cancer (%)	Other (%)
Parental care	8587	26.52	18.91	28.07	26.51
Residential care	88	35.23	18.18	18.18	28.41
Non-residential care	139	38.13	20.86	21.58	19.42
TOTAL N	8814	2361	1669	2456	2328

*ONS* Office of National Statistics.

**Fig. 1:**
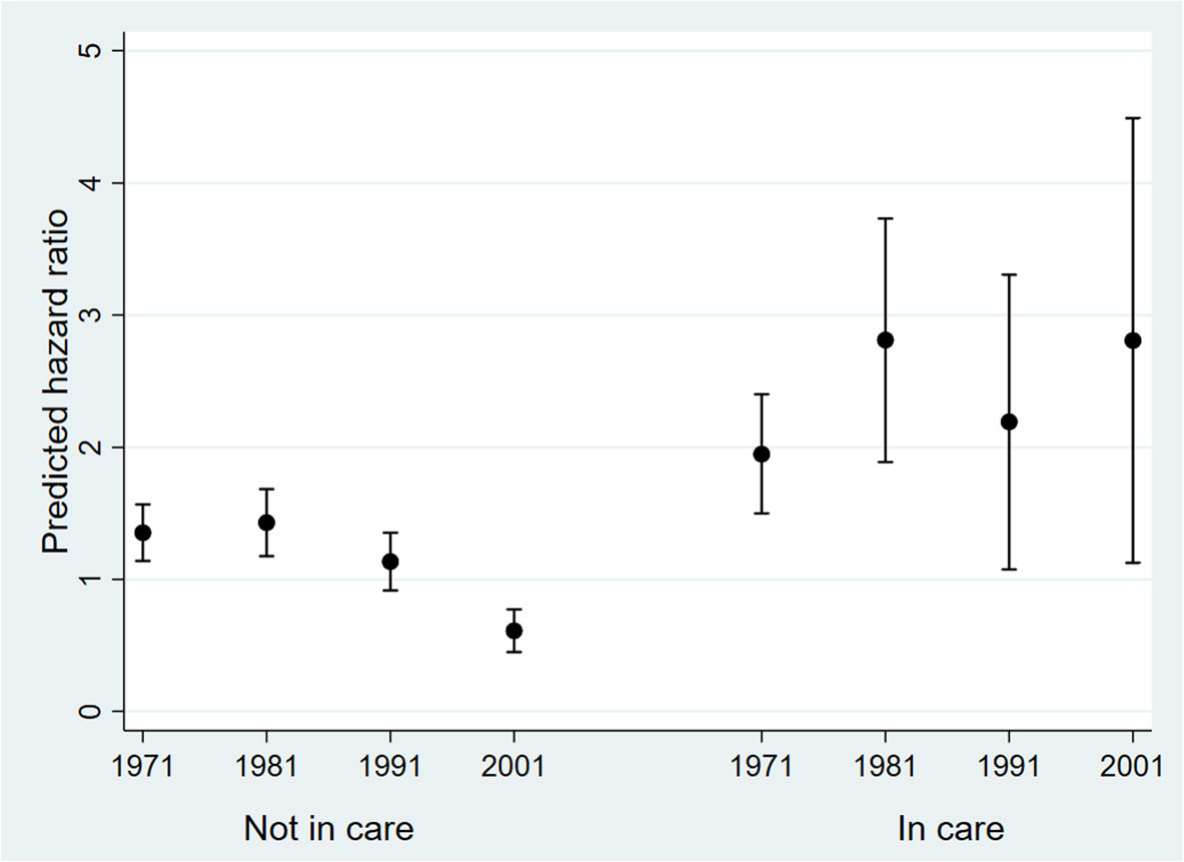
Predicted mortality hazard ratios for care status by baseline census year interaction, ONS Longitudinal Study. Adjusted for age and age^2^ at baseline, gender, country of birth and number of observations per individual

Those who had been in care in childhood were, at any given time point during the follow-up period, 70% more likely to die (Hazard ratio 1.70; 95% CI 1.49, 1.93) than those who had not been in care (Table 4, model 1). This was attenuated slightly with the addition of controls (model 2). There was no indication of an interaction between care status and gender (model 3, *p* = 0.49) and only weak support for a care status by baseline age interaction (model 4, *p* = 0.09). In the final model (Table 4, model 5), the association between care and all-cause mortality varied by baseline census year (*p* = 0.0009); for those first observed in the 1971 census, the hazard ratio for the care group was 1.44 (95% CI 1.23, 1.69) times that for those who had not been in care. When average hazard ratios were predicted for each care by baseline census year group (Fig. 1), mortality risk declined over time for those not in care but remained higher for care groups in all census years, especially for the 1981 and 2001 census years, resulting in 4.61:1 difference in mortality risk for those who had been in care versus not.

**Table 4:** Hazard ratios (95% confidence intervals) from Cox regression models of death by care status, ONS Longitudinal Study

	Model 1: Care status	Model 2: + Controls	Model 3: + Gender interaction	Model 4: + Age interaction	Model 5: + Baseline census interaction
Main effects					
In care	1.70 (1.49, 1.93)	1.63 (1.43, 1.86)	1.69 (1.43, 1.99)	2.01 (1.53, 2.65)	1.44 (1.23, 1.69)
Baseline age		0.97 (0.95, 0.99)	0.97 (0.95, 0.99)	0.97 (0.95, 0.99)	0.97 (0.95, 0.99)
Baseline age^2^		1.003 (1.002, 1.004)	1.003 (1.002, 1.004)	1.003 (1.002, 1.004)	1.003 (1.002, 1.004)
Male		1.64 (1.57, 1.71)	1.64 (1.57, 1.71)	1.64 (1.57, 1.71)	1.64 (1.57, 1.71)
Born outside UK		1.31 (1.17, 1.46)	1.31 (1.17, 1.47)	1.30 (1.16, 1.46)	1.31 (1.17, 1.47)
Baseline census					
1971 (ref)		1.00	1.00	1.00	1.00
1981		1.07 (1.00, 1.14)	1.07 (1.00, 1.14)	1.07 (1.00, 1.14)	1.06 (0.99, 1.13)
1991		0.84 (0.77, 0.93)	0.84 (0.77, 0.93)	0.85 (0.77, 0.93)	0.84 (0.76, 0.93)
2001		0.47 (0.40, 0.56)	0.47 (0.40, 0.56)	0.47 (0.40, 0.57)	0.45 (0.38, 0.54)
Number of observations					
One (ref)		1.00	1.00	1.00	1.00
Two		0.67 (0.62, 0.72)	0.67 (0.62, 0.72)	0.67 (0.62, 0.72)	0.67 (0.62, 0.72)
Interaction terms					
Care by male gender			1.10 (0.84, 1.44)		
Care by age				0.98 (0.96, 1.00)	
Care by baseline census					
In care in 1971 (ref)					1.00
In care in 1981					1.36 (0.99, 1.88)
In care in 1991					1.34 (0.81, 2.23)
In care in 2001					3.19 (1.74, 5.86)

*N* = 353,601, observations = 491,539.

### Supplementary analyses

Marginal predictions from the final model are first shown in Table 5 (model 5). When the reference group who had not been in care was confined to those in disadvantaged households only, there was minimal attenuation of the predicted hazard ratios (model SM1). This suggests the findings were robust to the choice of the comparison group.

**Table 5:** Marginal predictions of the mortality hazard ratios for all combinations of care status with baseline census year from sensitivity models, ONS Longitudinal Study

In care	Baseline census	Model 5	Model SM1 Disadvantaged non-care group	Model SM2 Residential care only	Model SM3 Non-residential care only	Model SM4 Deaths > 17 years old only
No	1971	1.35 (1.14, 1.57)	1.35 (0.92, 1.78)	1.30 (1.09, 1.51)	1.31 (1.11, 1.52)	2.95 (2.46, 3.44)
No	1981	1.43 (1.18, 1.68)	1.59 (1.02, 2.16)	1.38 (1.13, 1.63)	1.39 (1.14, 1.64)	3.27 (2.66, 3.88)
No	1991	1.13 (0.92, 1.35)	1.43 (0.87, 2.00)	1.09 (0.88, 1.31)	1.10 (0.89, 1.32)	2.47 (1.95, 2.99)
No	2001	0.61 (0.45, 0.77)	0.55 (0.22, 0.89)	0.59 (0.43, 0.74)	0.59 (0.44, 0.75)	1.72 (1.16, 2.28)
Yes	1971	1.95 (1.50, 2.40)	1.77 (1.12, 2.42)	3.36 (2.33, 4.39)	1.43 (1.06, 1.80)	4.15 (3.16, 5.13)
Yes	1981	2.81 (1.89, 3.73)	2.59 (1.41, 3.77)	7.24 (3.91, 10.57)	1.93 (1.16, 2.69)	6.25 (4.09, 8.40)
Yes	1991	2.19 (1.08, 3.31)	2.08 (0.83, 3.33)	4.15 (−0.68, 8.98)	1.92 (0.86, 2.99)	4.98 (2.21, 7.75)
Yes	2001	2.81 (1.13, 4.49)	2.70 (0.83, 4.57)	31.50 (−4.98, 67.97)	2.09 (0.66, 3.51)	9.05 (2.10, 16.00)
Wald test		16.42	13.11	30.14	12.71	13.59
p		0.0009	0.004	< 0.00005	0.005	0.004
N		353,601	81,967	350,477	353,090	353,601
Observations		491,539	93,567	485,770	490,627	491,539

When the reference group remained unchanged and instead the care group was confined to those who had been in residential care (model SM2), the predicted hazard ratios were amplified, consistent with the assumption that those in residential care had poorer health in childhood than those in non-residential care. This was most marked for the more recent baseline census years due to small cell sizes for the residential care by census year interaction estimates (Supplementary Table S2). When the reference group remained unchanged and the care group was confined to those who had been in non-residential care (model SM3), the predicted hazard ratios were attenuated but more similar to those observed for the final model. Nevertheless, despite no difference in the hazard ratios for the non-residential care group (1.43; 95% CI 1.06, 1.80) versus non-care group (1.31; 95% CI 1.11, 1.52) for those first observed in 1971, a significant difference over time emerged so that for those first observed in 2001 the predicted average hazard ratios were 2.09 (95% CI 0.66, 3.51) and 0.59 (95% CI 0.44, 0.75), respectively.

The full analysis sample was then reduced by removing any LS members who had died while still a child (i.e. < 18 years, model SM4). The predicted average hazard ratios were amplified but the general pattern of results remained the same despite few deaths among cohorts with shorter follow-up resulting in wider confidence intervals for these predictions.

## Discussion

### Statement of principal findings

In this large, nationally representative follow-up study of dependent children resident in England and Wales, consistent with hypothesis 1, all-cause mortality was higher among adults who had been in care up to 42 years earlier. There was no support for hypothesis 2; boys in care did not have a higher mortality risk than girls in care, nor for hypothesis 3 concerning an increased vulnerability if first observed in care at older ages. Finally, the reduction in mortality for those who were not in care across the census years 1971–2001 is not replicated for children who were in care; the difference in the mortality ratio for children in care was greater in 1981 and 2001 than in the 1971 census, contrary to hypothesis 4. The excess mortality for those who had been in care was mainly attributable to deaths categorised as self-harm, accidents and mental & behavioural causes.

### Results in relation to other studies

Our overall finding that being in care in childhood was associated with higher adult all-cause mortality is consistent with the few previous studies on this topic [3, 14–16]. We expand on most of these studies [14–16] by showing that the elevated risk of mortality is apparent long past early adulthood for some LS members. One UK study, using the same data set as us, had compared mortality rates 30 years after care assessment but was restricted to the 1971 baseline census year only, with care in residential facilities only [3]. This is an important restriction, as their data show a relative risk of 2.43 (our calculation) for those in residential care is substantively attenuated in our data (relative risk 1.62) based on all care experiences and the addition of more recent censuses.

Only two other studies we are aware of have investigated gender, timing of care experience and mortality [15, 16]. Although both studies stratified results by gender, neither reported statistical tests of gender differences in deaths among care leavers. However, it is clear that our results on gender concur with theirs in finding no gender differences overall.

Extrapolating from their results also suggests that the age in care with mortality relationship from these studies found a lower risk if in care aged 1–10 compared with being in care aged 11–17 years in the Finnish study [15], but a non-significantly lower mortality risk aged 0–6 year compared with aged 7–12 years first in care in the Swedish study [16]. Our estimated hazard ratio of 0.98 for the interaction between age and being in care disagrees with both prior studies but warrants replication before drawing any inferences. Nevertheless, differences in findings between the two studies and ours could be due to the different sampling procedures, or the longer follow-up period in our study, or to the greater degree of accuracy in their study about when placements began and ended, or to the other studies employing a categorical rather than linear age term.

As far as we are aware, we are the first study to show that the relationship between care status and mortality varied by census year, exemplifying the inverse care law [31].

### Implications and future research

The mechanism(s) by which being in care could be affecting mortality until later in adulthood is unclear. Numerous studies have shown that the mental and physical health, well known predictors of later mortality [32, 33], of children who enter care are worse than for the general population of children [5, 34–37]. This could be due to children with chronic conditions being more likely to be placed into care [32, 36, 37] or could reflect adverse effects of early life neglect and abuse [35, 38]. While we could not run the survival analysis for specific causes of death due to small numbers, descriptive data in our study showed the higher risk of mortality confined to ‘unnatural’ causes of death, rather than other categories, implicating mental ill-health as a key concern warranting further research. Furthermore, the finding of an increased risk of mortality when restricting the analysis to deaths in adulthood is consistent with the idea that children who have been in care find the transition to adulthood more difficult to negotiate [15]. A joint focus on physical and mental health, work, family and living arrangements at this life stage for care experienced individuals would be instructive.

Adults who have been in care are already known to have poorer social outcomes [3, 5, 8, 12, 39], with educational attainment, unemployment, unskilled occupation, homelessness and prison residence specifically known to be related to mortality [3, 40, 41]. One study examining mortality differences of Swedish 18-year olds who had been in foster care found that their mortality risk was similar to a comparison group that had been in contact with Welfare services, a group that should have had an equivalent distribution of social and health issues [16]. We found higher mortality risk for those who had been in care when a socially disadvantaged comparison group was used. Social determinants of health are also known to elevate cardiovascular and cancer mortality [42, 43], but this was not seen in our data. Taken together these findings suggest that it may not be the traditional social determinants that are driving the excess mortality risk, rather it could be a consequence of something specific to the need for care which is not being ameliorated by being cared for away from parents. If this is confirmed, then more upstream interventions are indicated.

Possible explanations for the differences in risk for care-experienced children across censuses are unclear. There was a suggestion that for the 1981 census, mortality was also higher for children who were not in care in childhood. Whatever mechanism was generally elevating mortality at that time, maybe entering the workforce during the 1990 and post-2008 recessions [44, 45], they could have been particularly challenging for children transitioning out of care during that time. Particularly for the 2001 census, there are concerns that reduced council funding due to austerity measures has resulted in reduced quality of care for looked-after children [46, 47], which could affect the mental health of these young people [48]. Alternatively, the elevated 2001 census mortality risk could be comparable to other censuses as they age, as a higher percentage of deaths occurred in the 20–40 year old age range for those who had been in care versus post-40 in the no care group; however when we restricted the follow-up to 13 years in all in census years, the excess 1991 and 2001 census mortality risk for children in care remained (HR 3.10 vs. 3.19 in Table 4).

By separately analysing residential and non-residential care experiences, we have a more nuanced understanding of the potential impact of being placed in care. With the reduction in relative risk of death and notwithstanding the higher hazard associated with non-residential care in 2001 (when the proportion of residential out-of-home care plummeted, Supplementary Table S2), this gives some support to the 1989 Children Act recommendation to give priority to relative care where possible.

### Strengths and limitations of the study

The main strength of this paper is its ability to follow-up a nationally representative group of English and Welsh children prospectively for up to 42 years after initial care assessment. This allowed us to investigate whether care status in childhood was associated with mortality decades after the children had left care. Combined with the additional strength of the LS regularly being linked to national death records, this meant that there was no loss to follow-up. The repeated data collections across five censuses also allowed us to assess whether associations have changed over time; a distinct possibility as policies related to looked-after children, and their transitions to adulthood, have changed over the four decades of this study.

The major disadvantage of using the LS dataset is that census data are only collected every 10 years and a limited range of relevant covariates was available to us. Moreover, as the data are from the census, we are unable to identify children with and without local authority care orders. As well, 61% of the sample only contributed information at one census during childhood, resulting in some imprecision concerning measurement and timing of the exact periods in which children were in care. Given the relatively young age of the sample at follow-up, and corresponding low percentage of deaths in the population, it is unlikely that missing census data was due to mortality. However, in our data children in care were more likely to be non-respondents to a preceding or proceeding census. A further limitation is the shorter follow-up period for children observed more recently, with a less than 13-year follow-up for those observed in the 2001 census year. This could have biased the care by census year interaction tests, most likely making differences conservatively estimated. Finally, lack of data on reason(s) for care placement and family characteristics prior to care, which are likely to correlate highly with mortality, point toward some residual confounding in our data.

## Conclusions

Adults who have been in care in childhood experience higher mortality risk after they have left care, especially to causes which can be ascribed as ‘unnatural deaths’. Further research should focus on the mechanisms underpinning these findings, particularly the role of mental health and why being in care during the early 1980s and 2000s is associated with excess mortality. If the findings reflect a true causal relationship between care and unnatural death in adulthood, current guidelines for transitions from child to adult health services [49] should be expanded to well beyond the initial young adult period.

## Supplementary information

**Additional file 1: Table S1**. Distribution of observations in childhood by census year, ONS Longitudinal Study. **Table S2**. Distribution of observations in out-of-home care by census year, ONS Longitudinal Study.

## Data Availability

The datasets generated and/or analysed during the current study are available to anyone in the UK who can fulfil the requirements of ONS’s Approved Researcher Scheme. The data can be accessed through the Secure Research Service (SRS) safe setting rooms at ONS offices. The application process is fully detailed on the CeLSIUS website at [www.ucl.ac.uk/celsius] where all the necessary forms can be found under the ‘Using the ONS Longitudinal Study’ section.

## Abbreviations

ANOVA: Analysis of variance
CeLSIUS: Centre for longitudinal study information & user support
CI: Confidence interval
LS: Longitudinal Study
ONS: Office for national Statistics
SIQR: Semi inter-quartile range
UK: United Kingdom
