# Supplementary material for: Association of childhood out-of-home care status with all-cause mortality up to 42-years later: Office of National Statistics Longitudinal Study
Source: BMC Public Health. 2020 May 20;20:735. doi: 10.1186/s12889-020-08867-3 (PMC7238620; doi:10.1186/s12889-020-08867-3)
Supplement: Supplementary file 1 — Additional file 1: Table S1. Distribution of observations in childhood by census year, ONS Longitudinal Study. Table S2. Distribution of observations in out-of-home care by census year, ONS Longitudinal Study. [file 12889_2020_8867_MOESM1_ESM.docx]

Table S1. Distribution of observations in childhood by census year, ONS Longitudinal Study

| Census | Baseline observation | Follow-up observation |
| --- | --- | --- |
|  | N | N |
| 1971 | 135,810 | 52,700^1^ |
| 1981 | 73,667 | 40,910 |
| 1991 | 74,440 | 44,328 |
| 2001 | 69,684 | 0^2^ |
| Total | 353,601 | 137,938 |

^1^ LS members’ 1^st^ observation in childhood left censored if before 1971

^2^ LS members’ 2^nd^ observation in childhood right censored if after 2001

Table S2. Distribution of observations in out-of-home care by census year, ONS Longitudinal Study

| Census | N | Residential care (%) | Non-residential care (%) |
| --- | --- | --- | --- |
| 1971 | 1,658 | 25.7 | 74.3 |
| 1981 | 2,347 | 12.3 | 87.7 |
| 1991 | 1,594 | 13.5 | 86.5 |
| 2001 | 2,063 | 4.0 | 95.9 |

Note: LS data can normally only be accessed in secure settings at ONS offices. From 18^th^ March 2020, the ONS Secure Research Service Safe Settings was closed because of the UK Government’s response to the COVID-19 pandemic. At the time of writing, it is not possible to extract the data for the table using the analysis sample in this paper. Instead, the data come from another analysis sample which had further exclusion criteria applied (7,639 observations, 97%).
